# Erosive tooth Wear in special Olympic athletes with intellectual disabilities

**DOI:** 10.1186/s12903-019-0727-3

**Published:** 2019-02-28

**Authors:** F. Marro, C. Fernandez, L. Martens, W. Jacquet, L. Marks

**Affiliations:** 1 Department of Paediatric Dentistry, PaeCoMeDiS research cluster, Ghent University, Ghent, Belgium; 2 Center of Special Care in Dentistry, PaeCoMeDiS, Ghent University, Ghent, Belgium; 3 Oral Health Research Group ORHE, Faculty of Medicine and Pharmacy, Vrije Universiteit Brussel, Brussels, Belgium; 4 Department of Educational Sciences EDWE-LOCI, Faculty of Psychology and Educational Sciences, VUB Vrije Universiteit Brussel, Brussels, Belgium; 5 Department Paediatric Dentistry & Special Care Dentistry, Dental School, Faculty of Medicine and Health sciences, University Gent, Gent, Belgium

**Keywords:** Erosive tooth wear, Patients with intellectual disability, Down syndrome, Special Olympics athletes

## Abstract

**Background:**

Special Olympics (SO) events represent an opportunity to obtain considerable information regarding intellectual disable (ID) patients. Studies done with SO data have shown an overview of the oral health status of these athletes; however, no information exists regarding the erosive tooth wear (ETW). Therefore, the aim of this study is to determine the presence and severity of ETW in athletes with ID who participated in the SO Belgium 2016.

**Methods:**

The study population consisted in 232 athletes with ID who participated in the SO special smiles program, Belgium 2016. For analysis, the sample was divided in three groups: a) athletes with ID under the age of 25 not diagnosed with Down Syndrome (DS) (*n* = 174), b) athletes with DS under the age of 25 (*n* = 39) and c) athletes with DS from 25 and older ages (*n* = 58). Two calibrated dentists performed dental examinations using the Basic Erosive Wear Examination Index (BEWE). The BEWE sum > 0 was used to determine prevalence of ETW. Severity was determined by two- indicators: 1) By risk levels (low, medium and high risk) proposed by the BEWE index, and 2) by the highest score reached per subject in at least one tooth (BEWE1, 2 or 3). Chi-square test and Mann-Whitney U test were used to detect significant differences among different groups (*p* < 0.05).

**Results:**

The prevalence of ETW for young athletes with ID was 51.14%. Within these athletes, the DS group presented a significant higher mean BEWE sum (4.67, SD 5.64) and prevalence of ETW (69.2%BEWE> 0) when compared to athletes without DS (mean BEWE sum: 1.96, SD 3.47 and 46.3% BEWE> 0; *p* < 0.05). Furthermore, a significantly higher percentage of athletes with DS were considered at high risk of ETW (p < 0.05).

**Conclusions:**

As a conclusion, half of the young athletes with ID presented at least one affected surface with ETW. The recorded prevalence and severity of ETW for the younger group of athletes with DS was distinctly higher than the athletes with ID not having DS. This shows the need to generate knowledge in order to provide correct management and prevention of erosive tooth wear in populations with ID.

## Background

Erosive tooth wear (ETW), known as the chemical-mechanical process of tooth wear caused principally by extrinsic/intrinsic acids [1], has become a topic of concern for the dental community. The apparent increase in the prevalence of ETW and the current dietary habits involving a high intake of extrinsic acids are the reasons behind this concern [1, 2]. In 2015, the worldwide prevalence of ETW was estimated to be 30% for children and adolescents [3], and in Europe a multicentre investigation suggested that 57% of the young adults (18–35 years old) have at least one affected surface by ETW [4]. Up to date, several studies have investigated the prevalence of ETW among the general population; however, there is limited evidence regarding how this condition affects minority groups such as the people with Intellectual disabilities (ID) [5–7].

In the past few years, the Special Smiles program organized by the Special Olympics contest (SOSS) has become an important platform to investigate and evaluate the oral health status and treatment needs of a considerable number of patients with ID [8]. This program collects data of athletes with ID from all over the world in order to understand their treatment needs and improve their access to oral health care [9]. Numerous publications using SOSS data suggest that patients with ID participating in such contest have a higher prevalence of periodontal diseases, poorer oral hygiene and higher rates of untreated decay when compared with the general population [8, 10]. Until now, several oral health parameters are included in the Special smiles screenings with much attention drawn in to the major oral health problems such as caries and periodontal diseases; however, other prevalent conditions affecting the oral health status have not been included.

Since the topic of ETW has become relevant over the past few years, it is highly important to include the study of this condition during SOSS events.

Therefore, the primary aim of the study was to include for the first time at SOSS event an examination of ETW in order to determine the prevalence and severity of ETW in a young group of athletes with ID (up to 25-yr-olds) participating in the SOSS, Belgium 2016. In addition, since one of the most prevalent syndromes presenting ID at SO events is Down Syndrome (DS), the secondary aim was to determine the same parameters on the entire group of athletes with Down syndrome (DS; all ages).

## Materials and methods

### Ethical issues

For this cross-sectional study, data were collected at the annual SO event held in May 2016 in La Louviere, Belgium. Ethical approval was obtained from the local ethical committee of the Ghent University Hospital according to the “ICH Good Clinical Practice” of the declaration of Helsinki (2016/0461). Participation consent from the athlete and/or the legal guardian was collected.

### Population

All the young athletes with ID (up to age 25, age range 9–25) and the older group of athletes diagnosed with DS (age 26 and up, age range 26–62) who participated in the SOSS program, Belgium 2016, were included in this study. Exclusion criteria were subjects who declined participation when they registered for the screening.

### Calibration of the examiners

Prior to the study, two examiners (FM and CF) were trained and calibrated during a period of two months against an experienced benchmark (LM) for the consistent diagnosing of ETW and the use of the BEWE index according to Bartlett et al. 2008 [11]. Intra-examiner reliability (acc. Landis and Koch) was substantial (0.78 for FM and 0.88 for CF) and the Inter-examiner reliability was 0.65 (unweight Cohen’s kappa).

### Clinical examinations

Intra-oral examinations were performed during the SOSS event. Standard illumination (LED head lamps) and sterile number 5 mouth mirrors were used to examine all surfaces of the permanent dentition. Before scoring, dental surfaces were dried using sterile compresses. Presence and severity of ETW lesions were recorded using the BEWE score criteria. According to this classification, every permanent tooth surface was examined and classified into 4 score criteria as follows: ‘0-’ an indication for absence of ETW, ‘1-’ an indication for initial loss of surface texture (visually detectable), ‘2’*- an indication for distinct defect of hard tissue loss with less than 50% of the surface area affected, and ‘3’*- an indication for hard tissue loss equal or more than 50% of the surface area (*can also involve dentin).

The “BEWE sum” calculated per individual was the result of the sum of the highest scores recorded per sextant as suggested by Bartlett et al. 2008 [11]. Severity and treatment strategies were determined by two- indicators: 1) By risk levels (low, medium and high risk) with the BEWE sum cut-offs proposed by the same author [11], and 2) By the highest score reached per subject in at least one tooth (BEWE = 1, 2 or 3). To avoid any interpretation bias, the book from Lussi & Jaeggi where the clinical use of BEWE index for dental erosion is graphically explained, was used as guidance during the entire study [12].

The presence of big restorations or appliances that did not allow, or compromised the quality of the scoring excluded the tooth. Moreover, buccal/ facial, occlusal, and lingual/palatal surfaces that presented tooth wear with a clear absence of acidic influence was scored as 0. Examples of this were pure attrition flat surfaces with correspondent antagonist, bruxism without any roundness or presence of cupping on occlusal surfaces, or pure abfraction on buccal surfaces and clear TW caused by foreign objects.

### Data management

Data were analysed with the IBM SPSS v. 22.0 (SPSS Inc., Armonk, NY) software. In order to fulfil the aims, data analysis was done at three levels. First, an analysis was conducted for the entire young group of athletes with ID up to age 25 to identify the overall prevalence, severity and levels of risk with respect to ETW. Second, the Mann Whitney U test was used to detect significant differences between the young athletes diagnosed with DS and the rest of the younger athletes with ID. Finally, in a third phase, athletes over the age of 25 diagnosed with DS together with the young athletes with DS were analysed in order to determine the presence of ETW in this specific group of athletes. For the entire analysis, Pearson Chi-square was used to detect significant differences among variables with a significance level of alpha =0.05.

## Results

### Descriptive data

A total of 723 athletes with ID participated in the SOSS program, Belgium 2016. From these, 232 (32.1%; mean age 23.9 ± 9.7; age range 9–62 years old) fulfilled the inclusion criteria and were effectively recruited for the study. The final sample obtained for the young athletes with ID was 174, with 22.4% of them being athletes with DS (*n* = 39). The remaining 58 participants were adult athletes diagnosed with DS (Table 1). Age and gender did not show significant differences in the young group of athletes.

**Table 1:** Prevalence of ETW and mean BEWE sum in Special Olympic athletes

		Age Mean ± SD	*P*-value	BEWE sum Mean ± SD	*P*-value	ETW Prevalence (BEWE sum> 0)	*P*-value
Young Athletes with ID *n* = 174	Athletes without DS *n* = 135	19.1 ± 3.5 Age range (9–25)	0.39	1.96 ± 3.47	< 0.005^*^	46.3%	< 0.05^*^
	Athletes with DS *n* = 39	19.5 ± 3.8 Age range (10–25)		4.67 ± 5.64		69.2%	
	Total	19.3 ± 3.5		2.52 ± 4.04		51.2%	
Older athletes with DS *n* = 58		37.7 ± 8.9		6.83 ± 4.57		94.8%	
		Age range (26–62)					

Comparative results were only performed between the young athletes with ID, which do not differ significantly in age. Mann-Whitney u test was used at significance level of p < 0.05 (*) for comparisons between mean BEWE sum and Chi ^*2*^ *Test was used to compare prevalence of ETW (p < 0.05)*

From all the participants in the SOSS program, only three refused dental examination due to anxiety reasons related with fear.

### ETW in young athletes with ID

Overall, the prevalence of ETW (BEWE sum > 0) for the young group of athletes with ID was 51.2% (Table 1).

Table 2 illustrates the ETW classification of athletes according to the highest severity reached in at least one tooth. Results indicate that 10.46% (*n* = 18) of athletes were scored BEWE = 3.

**Table 2:** Distribution of Special Olympics athletes according to the highest BEWE score obtained

Criteria	Young ID Athletes without DS *n* = 135	Young ID Athletes with DS *n* = 39	Older athletes with DS *n* = 58
BEWE = 0	72(53.33%)	12(30.77%)	3(5.17%)
BEWE = 1	41(30.37%)	12(30.77%)	15(25.86%)
BEWE = 2	10(7.40%)	9(23.07%)	20(34.48%)
BEWE = 3	12(8.88%)	6(15.38%)	20(34.48%)

*Athletes were distributed according the worst BEWE score obtained in at least one tooth*

**Fig. 1:**
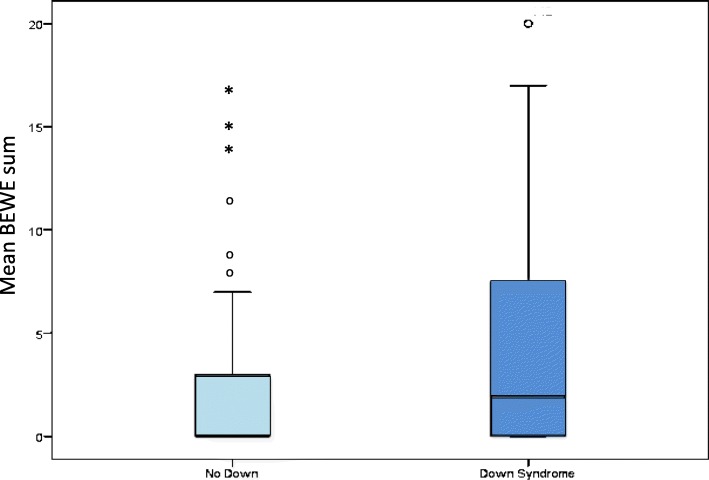
Mean BEWE sum scores for young athletes with ID with and without Down syndrome (up to 25-year-old)

Comparisons between young athletes with DS and those not having DS showed that mean BEWE sum scores (*p* < 0.005) and prevalence of ETW (*p* < 0.05) were significantly higher for young athletes with DS (Table 1 and Fig. 1).

**Fig. 2:**
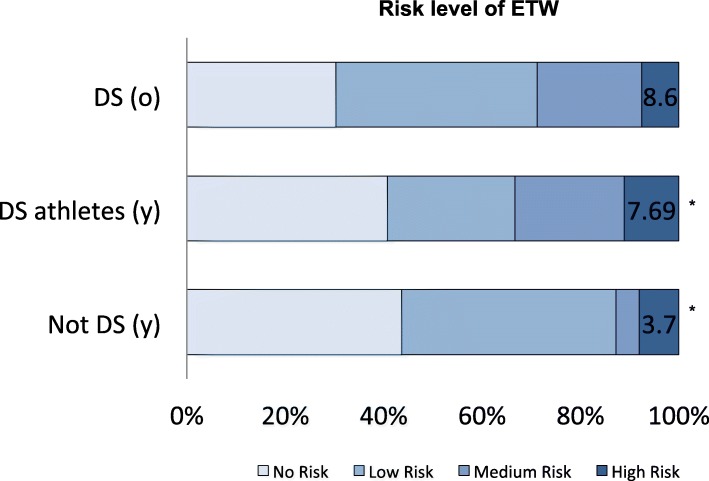
ETW risk in athletes with ID according to cut-offs values of Bartlett et al. 2008. No risk: BEWE sum > 0 up to 2; Low risk: BEWE sum > 2 up to 8; Medium risk: BEWE sum > 8 up to 13; High risk: BEWE sum > 13 up to 18. *P < 0.05 Pearson Chi-Square comparing risk differences between young groups of athletes with ID

Furthermore, a significantly higher percentage of athletes with DS were considered within the higher risk levels of ETW (p < 0.05; Fig. 2). The maximum BEWE sum reached was 17 in only one subject diagnosed with DS (age 19-year-old).

### ETW in the group of young and adults athletes with DS

The prevalence and mean BEWE sum of ETW for the group of athletes over age 25 with DS (*n* = 58) was 94.8% (BEWE> 0) and 6.83 (SD 4.6), respectively (Table 1). Only 3 subjects were free from ETW and the classification of the athletes according to the highest BEWE score reached showed that 34.5% had the most severe form in at least one tooth (Table 2).

## Discussion

To the best of our knowledge, this is the first study evaluating the presence of ETW in athletes with ID participating at the SOSS. The main result of this study show that approximately one half of the athletes with ID up to age 25 who were screened in Belgium have at least one tooth surface affected by ETW (51.2% BEWE sum > 0). Moreover, 10.46% (*n* = 18) of the athletes with ID had at least one tooth affected with the most severe form of ETW (BEWE = 3 at tooth level) and 4.59% of them were considered at high risk (BEWE sum > 13) according to the risk scale provided by Bartlett et al., 2008 [11].

The prevalence of ETW found in this study is within the range reported by several studies using BEWE index in young non-ID populations (between 15 to 79% BEWE sum > 0 for 12 to 15 yr-olds) [13–19]. However, interestingly athletes with ID duplicated the percentage of severe forms of ETW when compared to general population, where often low severity rates are found [20, 21]. More precisely, the majority of the studies report that less than 5% of the subjects reach BEWE = 3 in at least one tooth (the most severe form) or that BEWE =3 scores are hardly found in the screened populations [16, 17, 21]. The fact that around 10% of the young athletes with ID in this study were in need of a restorative treatment due to ETW reasons differs clearly with the aforementioned data and may indicate that this group is at higher risk of ETW.

It appears that the athletes having DS are responsible for the higher severity of ETW found in this study (Table 1). Young athletes with DS have shown significantly more severe forms of ETW when compared with the young athletes with ID not having DS. Additionally, the older group of athletes with DS presented high prevalence rates (94.8%) and severe forms of ETW, which differ considerably from the general population. This outcome is similar to the results shown by Bell et al. [7], where a group of people with DS had a significant higher index of TW (59.2% pathological TW and 34.7% severe TW) in comparison to a control group. Nevertheless, caution should be taken at the moment to compare both studies since the present study specifically aimed to assess ETW.

Some medical and orofacial characteristics linked with DS patients may help explain the high severity of ETW found. These include among others the presence of reflux or GERD (Gastroesophageal Reflux Disease), use of medications that induce xerostomia, malocclusions, mouth breathing and a higher prevalence of bruxism [7, 22–24]. GERD has been several times recognized as risk factor for ETW [25], and in the case of DS literature suggests that around 13.8 to 59% of them suffer from this kind of gastric disorders [26]. It is possible that the combination between GERD and bruxism exacerbates the severity of ETW in these patients. This is consistent with the literature where it is suggested that TW processes do not occur in isolation and they are rather a result of multiple interactions [27]. This hypothesis may thus be accepted; however, the lack of evidence aiming to investigate the reasons behind the severe cases of ETW in DS patients indicates the need for further studies.

The results of this study should be interpreted within the limitations discussed in previous studies performed with SOSS populations. The sample obtained at SOSS events does not represent the entire population of people with ID for different reasons. This group of athletes with ID are well supported by their families and may receive frequent medical and dental care [28] and are very involved in sports. The exclusive selection of the younger group of athletes (up to 25 years of age) which, according to previous SOSS data, annually represents 26.9% (*n* = 169) of the total number of athletes screened could be a bias [9]. On the other hand, the entire group of athletes with DS, was selected in order to obtain an idea of the oral status of this prevalent syndrome.

The study aimed to detect prevalence and severity of ETW to provide an idea of the oral status with respect to ETW in athletes with ID. Although the literature suggests that the application of questionnaires to assess the etiological factors is crucial for studies regarding ETW/TW, the detection of possible etiological factors through the use of a questionnaire was not performed, principally due to the different levels of comprehension related to the condition of the athletes with ID. Moreover, most of the athletes were accompanied by a trainer who is not well informed about the personal habits of the athlete. Questionnaires targeting caregivers could be useful for further elucidation of the potential aetiology of ETW in this population.

## Conclusions

As a conclusion, half of the young athletes with ID presented at least one affected surface with ETW; however, the recorded prevalence and severity of ETW for the younger group of athletes with DS was distinctly higher than the athletes with ID not having DS. Furthermore, the severity reached by the DS group of athletes differs from the low severity of ETW commonly presented in the majority of the studies performed in general populations. This shows the need to generate knowledge with respect to aetiological factors involved in this specific population, in order to provide a correct management and prevention of ETW in populations with ID.

## Abbreviations

BEWE: Basic erosive wear examination
DS: Down syndrome
ETW: Erosive tooth wear
ID: Intellectual disable
SO: Special Olympics
SOSS: Special Olympics special smiles program.
TW: Tooth wear
